# Identification of known and novel pancreas genes expressed downstream of Nkx2.2 during development

**DOI:** 10.1186/1471-213X-9-65

**Published:** 2009-12-10

**Authors:** Keith R Anderson, Peter White, Klaus H Kaestner, Lori Sussel

**Affiliations:** 1 Department of Biochemistry and Program in Molecular Biology, University of Colorado Health Science Center, Denver, CO, 80045, USA; 2 Department of Genetics and Development, Columbia University, New York, NY 10032, USA; 3 Department of Genetics and Institute for Diabetes, Obesity and Metabolism, University of Pennsylvania, 752B CRB, 415 Curie Blvd, Philadelphia, Pennsylvania 19104, USA; 4 The Research Institute at Nationwide Children's Hospital, 700 Childrens Drive, Columbus, OH 43205, USA

## Abstract

**Background:**

The homeodomain containing transcription factor Nkx2.2 is essential for the differentiation of pancreatic endocrine cells. Deletion of Nkx2.2 in mice leads to misspecification of islet cell types; insulin-expressing β cells and glucagon-expressing α cells are replaced by ghrelin-expressing cells. Additional studies have suggested that Nkx2.2 functions both as a transcriptional repressor and activator to regulate islet cell formation and function. To identify genes that are potentially regulated by Nkx2.2 during the major wave of endocrine and exocrine cell differentiation, we assessed gene expression changes that occur in the absence of Nkx2.2 at the onset of the secondary transition in the developing pancreas.

**Results:**

Microarray analysis identified 80 genes that were differentially expressed in e12.5 and/or e13.5 Nkx2.2^-/- ^embryos. Some of these genes encode transcription factors that have been previously identified in the pancreas, clarifying the position of Nkx2.2 within the islet transcriptional regulatory pathway. We also identified signaling factors and transmembrane proteins that function downstream of Nkx2.2, including several that have not previously been described in the pancreas. Interestingly, a number of known exocrine genes are also misexpressed in the Nkx2.2^-/- ^pancreas.

**Conclusions:**

Expression profiling of Nkx2.2^-/- ^mice during embryogenesis has allowed us to identify known and novel pancreatic genes that function downstream of Nkx2.2 to regulate pancreas development. Several of the newly identified signaling factors and transmembrane proteins may function to influence islet cell fate decisions. These studies have also revealed a novel function for Nkx2.2 in maintaining appropriate exocrine gene expression. Most importantly, Nkx2.2 appears to function within a complex regulatory loop with Ngn3 at a key endocrine differentiation step.

## Background

The pancreas is a multifunctional organ that is critical for maintaining glucose homeostasis and for producing many of the enzymes required for digestion of carbohydrates, lipids and proteins. To carry out these diverse functions, the pancreas contains three major tissue types: the exocrine acinar cells, the endocrine cells that comprise the islet of Langerhans, and the ductal epithelial cells. Although each of these pancreatic components performs unique functions, all are derived from a defined set of endodermally-derived progenitors [1]. Subsequent pancreatic morphogenesis and differentiation of these progenitor populations is dependent on the concerted action of multiple transcriptional regulators. Early during pancreatic bud evagination, Pancreatic duodenal homeobox 1 (Pdx1) and Pancreatic transcription factor 1a (Ptf1a) are co-expressed in the pancreatic progenitor population [1,2]. Ptf1a, a basic helix-loop-helix (bHLH) transcription factor, becomes restricted to the exocrine cell population, where it is essential for exocrine cell formation and function [2,3]. Pdx1 expression is maintained throughout the early pancreatic epithelium and becomes mostly restricted to β and δ cells after the secondary transition, although low levels of Pdx1 persist in some acinar cells into adulthood [4-6]. Pdx1 has distinct functions at each developmental stage and in each of the cell types where it is expressed, and itself is critically dependent on the winged helix transcription factors Foxa1 and Foxa2 [7-9]. Ngn3, a bHLH transcription factor, is required downstream of Pdx1 to activate the endocrine differentiation program [10-13]. Further islet cell fate determination in the Ngn3^+ ^cells then depends on a number of additional transcription factors including Pdx1, Nkx2.2, Pax4, Pax6, Isl1, NeuroD1, Arx, and Nkx6.1, each of which has been identified and characterized through genetic deletion or overexpression studies [14-19]. These and other transcription factors are then necessary for appropriate neogenesis, differentiation, and maturation of the five islet cell types (recently reviewed in [5,20-22]).

Although much is known about the identity, expression and function of many of the intrinsic regulatory proteins involved in pancreatic development and differentiation, the identification of downstream targets of these transcription factors to mediate these developmental events has proven to be more challenging. In efforts to identify downstream functional players during pancreagenesis, multiple groups have determined pancreatic gene expression profiles of wild type and transcription factor knockout models, including Pdx1, Ngn3, NeuroD1, and FoxA2 [23-30]. Alternatively, temporal comparisons between transcription factor positive cell populations, such as Pdx1^+ ^or Ngn3^+ ^[31,32], by microarray analysis have also uncovered important downstream factors. Here we present the first study comparing gene expression alterations between wild type and Nkx2.2^-/- ^mouse pancreata at e12.5 and e13.5, the stages marking the onset of the secondary transition when the major wave of islet cell type specification events occur for both endocrine and exocrine pancreas.

Nkx2.2 is a homeodomain containing transcription factor critical for endocrine pancreas specification [19]. Prior to e15.5, the pancreas of Nkx2.2^-/- ^mice appears overtly normal and contains wild type numbers of undifferentiated Pdx1+ pancreatic epithelium and Ngn3+ endocrine progenitor cells [12,19,33]. As development proceeds, the Nkx2.2^-/- ^mice form the normal numbers of total endocrine and exocrine cells; however, within the endocrine compartment all β cells, most α cells and a subset of PP cells fail to differentiate and are replaced by a ghrelin-producing cell population, which persists to comprise the majority of a misshapen islet. Nkx2.2^-/- ^mice die within days after birth with severe hyperglycemia [19,34]. Nkx2.2 expression is initiated at e8.75 in the dorsal pancreas and e9.5 in the ventral pancreas, shortly after the activation of Pdx1 in each anlage [5]. High level Nkx2.2 expression subsequently becomes restricted to the centralized epithelium at e11.5, where it is upregulated in a large number of the Ngn3^+ ^endocrine progenitor cells [5,12]. Cells expressing low levels of Nkx2.2 are also apparent throughout the undifferentiated pancreatic epithelial population until after the secondary transition [5]. Multiple *in vitro *and *in vivo *studies suggest that Nkx2.2 mediates early islet cell fate decisions by functioning both as a transcriptional repressor and activator, depending on temporal and cellular context [35-39]. To date, however, MafA and insulin (Ins2) are the only two verified direct targets identified downstream of Nkx2.2 in the pancreas [35,38]. MafA and Ins2 both represent mature β cell genes; the molecular mechanisms by which Nkx2.2 controls early endocrine specification remain largely unknown. In an effort to identify downstream direct and indirect targets of Nkx2.2 that promote or inhibit appropriate islet cell differentiation, we performed microarray analyses at e12.5 and e13.5 during pancreas development. This analysis has resulted in the identification and characterization of expression differences for several known and previously uncharacterized pancreatic genes. These studies clarify the position of Nkx2.2 within the known transcription factor network that regulates islet specification. Furthermore, we have identified a novel role for Nkx2.2 in the regulation of several exocrine-specific genes, suggesting that Nkx2.2 may also function to influence gene expression in the early Pdx1^+ ^Ptf1a^+ ^pancreatic progenitor cell.

## Results and Discussion

### Gene expression differences between wild type and Nkx2.2^-/- ^pancreata

To identify genes that function downstream of Nkx2.2 during early pancreas development, we compared gene expression profiles of e12.5 or e13.5 wild type and Nkx2.2^-/- ^pancreata. We restricted our analysis to the onset of the secondary transition, when the principal wave of endocrine differentiation begins, to enrich for downstream direct and indirect targets of Nkx2.2 that are involved in the early events of islet cell type specification. Global gene profiling at these time points also allowed us to minimize gene expression differences caused by the islet cell type conversions that occur later in the Nkx2.2^-/- ^mice. In addition, between e12.5 and e13.5, Nkx2.2 is expressed broadly throughout the undifferentiated pancreatic epithelium, which is present in comparable amounts between wild type and Nkx2.2^-/- ^pancreata (Additional File 1: Figure S1 and data not shown; [33]). This allows for the enrichment of equal quantities of wild type and mutant tissue that would normally contain a high percentage of Nkx2.2-expressing cells.

**Figure 1:**
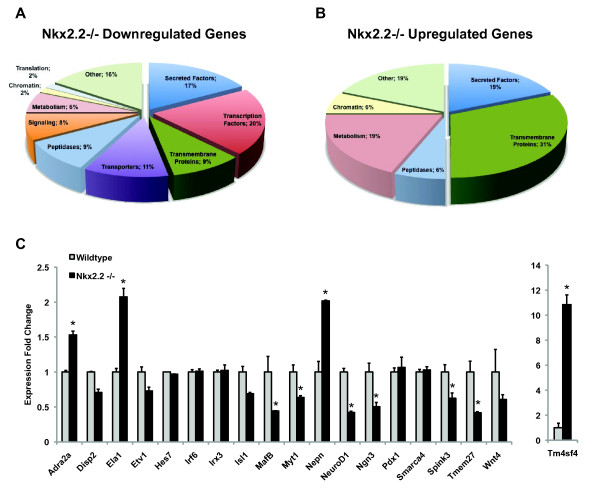
**Categorization and confirmation of Nkx2.2^-/- ^gene expression changes**. (A, B) Gene changes categorized by gene ontology (GO) terms determined in Tables 1, 2, and 3. Quantification of downregulated (A) and upregulated (B) genes is represented as percentages when compared to total affected genes. (C) qRTPCR verification of genes of interest at e13.5. Genes are listed in alphabetical order, except Tm4sf4, which was graphed separately due to a larger y-axis data range. Error bars represent SEM and asterisks denotes statistical significance < 0.05. Wild type = grey bars, Nkx2.2^-/- ^= black bars.

Pools of cDNA generated from e12.5 and e13.5 pancreata (n = 5 per stage, per genotype) were amplified, labeled and hybridized to PancChip 6.1 (UPenn) microarrays containing 13,059 mouse cDNAs [32]. Both statistical significance (<20% FDR) and a fold change threshold of 1.5 were used to select genes for further verification and analysis. At e12.5, 65 genes were differentially expressed, of which 49 were downregulated and 16 upregulated in Nkx2.2^-/- ^pancreata compared to wild type (Table 1 and 2). By e13.5, an additional 15 genes were downregulated, which may begin to reflect the differences in islet cell type populations between wild type and Nkx2.2^-/- ^mice and/or may be due to the identification of additional direct or secondary downstream Nkx2.2 targets (Table 3). We used gene ontology (GO) terms to categorize the protein products of genes misregulated in the Nkx2.2^-/- ^pancreas (Table 1, 2, and 3, Figure 1A, B). Many of the genes encode proteins not previously identified in the pancreas, as well as known pancreatic factors. As expected, we saw a large decrease in Nkx2.2 expression, as well as significant changes in the expression of glucagon, insulin and ghrelin (Table 1, 2, and 3). As further validation of the experimental approach and microarray platform, we used quantitative real-time PCR to verify many of the altered genes that represented several classes of proteins. Of those tested, 77.8% displayed expression changes that corresponded well with the microarray analysis (Figure 1C), closely reflecting the 20% FDR used as a statistical cutoff. Below we discuss the further characterization of a select subset of genes with altered expression using quantitative real-time PCR (qRTPCR) to compare their gene expression levels and mRNA *in situ *analysis to compare their spatial localization in wild type versus Nkx2.2^-/- ^pancreata.

**Table 1:** Downregulated genes in Nkx2.2^-/- ^versus Nkx2.2^+/+ ^pancreata at e12.5.

Genbank Accession Number	*Gene Symbol*	Gene Name	e12.5 Fold Change
**Secreted Factors**			
NM_008100	*Gcg*	GLUCAGON	-20.0
NM_145435	*Pyy*	PEPTIDE YY	-7.6
NM_007694	*Chgb*	CHROMOGRANIN B	-2.0
NM_009242	*Sparc*	SECRETED ACIDIC CYSTEINE RICH GLYCOPROTEIN	-1.9
NM_009258	*Spink3*	SERINE PEPTIDASE INHIBITOR, KAZAL TYPE 3	-1.8
NM_010257	*Gast*	GASTRIN	-1.7
NM_008343	*Igfbp3*	INSULIN-LIKE GROWTH FACTOR BINDING PROTEIN 3	-1.6
NM_008386	*Ins1*	INSULIN I	-1.5
			
**Transcription Factors**			
NM_010919	*Nkx2-2*	NK2 TRANSCRIPTION FACTOR RELATED, LOCUS 2 (DROSOPHILA)	-5.0
NM_009719	*Neurog3*	NEUROGENIN 3	-2.7
NM_025824	*Bzw1*	BASIC LEUCINE ZIPPER AND W2 DOMAINS 1	-1.9
NM_010894	*Neurod1*	NEUROGENIC DIFFERENTIATION 1	-1.9
NM_033041	*Hes7*	HAIRY AND ENHANCER OF SPLIT 7 (DROSOPHILA)	-1.8
NM_008092	*Gata4*	GATA BINDING PROTEIN 4	-1.7
NM_178083	*Irf6*	INTERFERON REGULATORY FACTOR 6 V-MAF MUSCULOAPONEUROTIC FIBROSARCOMA ONCOGENE	-1.6
NM_010658	*Mafb*	FAMILY, PROTEIN B (AVIAN)	-1.6
NM_019738	*Nupr1*	NUCLEAR PROTEIN 1	-1.6
			
**Transmembrane Proteins**			
NM_020626	*Tmem27*	TRANSMEMBRANE PROTEIN 27	-4.5
NM_011695	*Vdac2*	VOLTAGE-DEPENDENT ANION CHANNEL 2	-2.8
NM_011150	*Lgals3bp*	LECTIN, GALACTOSIDE-BINDING, SOLUBLE, 3 BINDING PROTEIN	-1.6
NM_144926	*Sez6l2*	SEIZURE RELATED 6 HOMOLOG LIKE 2	-1.6
			
**Transporters**			
NM_013697	*Ttr*	TRANSTHYRETIN	-3.6
NM_172479	*Slc38a5*	SOLUTE CARRIER FAMILY 38, MEMBER 5	-3.2
NM_007618	*Serpina6*	SERINE (OR CYSTEINE) PEPTIDASE INHIBITOR, CLADE A, MEMBER 6	-1.7
NM_007423	*Afp*	ALPHA FETOPROTEIN	-1.6
NM_007443	*Ambp*	ALPHA 1 MICROGLOBULIN/BIKUNIN	-1.6
			
**Peptidases**			
NM_008792	*Pcsk2*	PROPROTEIN CONVERTASE SUBTILISIN/KEXIN TYPE 2	-2.2
NM_025583	*Ctrb1*	CHYMOTRYPSINOGEN B1	-1.8
NM_001024698	*Cpa2*	SIMILAR TO CARBOXYPEPTIDASE A2 PRECURSOR	-1.7
NM_011909	*Usp18*	UBIQUITIN SPECIFIC PEPTIDASE 18	-1.6
NM_013494	*Cpe*	CARBOXYPEPTIDASE E	-1.6
NM_146101	*Habp2*	HYALURONIC ACID BINDING PROTEIN 2	-1.6
			
**Signaling**			
NM_021356	*Gab1*	GROWTH FACTOR RECEPTOR BOUND PROTEIN 2-ASSOCIATED PROTEIN 1	-2.5
NM_145736	*Pim2*	PROVIRAL INTEGRATION SITE 2	-1.7
NM_009245	*Serpina1c*	SERINE (OR CYSTEINE) PEPTIDASE INHIBITOR, CLADE A, MEMBER 1A	-1.6
NM_008052	*Dtx1*	DELTEX 1 HOMOLOG (DROSOPHILA)	-1.5
			
**Metabolism**			
NM_018874	*Pnliprp1*	PANCREATIC LIPASE RELATED PROTEIN 1	-2.5
NM_001081070	*Pdia2*	PROTEIN DISULFIDE ISOMERASE ASSOCIATED 2	-2.1
NM_009040	*Rdh16*	RETINOL DEHYDROGENASE 16	-1.5
			
**Translation**			
NM_008774	*Pabpc1*	POLY A BINDING PROTEIN, CYTOPLASMIC 1	-1.5
			
**Other**			
CV676831	*9330162012Rik*	CDNA RIKEN 9330162012 GENE	-3.3
CF585856	*IMAGE = 6430431*	Unknown	-3.3
NM_026791	*Fbxw9*	F-BOX AND WD-40 DOMAIN PROTEIN 9	-2.8
CF582515	*IMAGE = 6432606*	Unknown	-2.6
NM_026479	*Zcchc10*	ZINC FINGER, CCHC DOMAIN CONTAINING 10	-2.0
NM_010058	*Dmwd*	DYSTROPHIA MYOTONICA-CONTAINING WD REPEAT MOTIF	-1.9
NM_134013	*Psme4*	PROTEASOME (PROSOME, MACROPAIN) ACTIVATOR SUBUNIT 4	-1.6
NM_198000	*1700001O22Rik*	RIKEN CDNA 1700001O22 GENE	-1.6
NM_025946	*2010100O12Rik*	RIKEN CDNA 2010100O12 GENE	-1.6

49 genes were downregulated in Nkx2.2^-/- ^pancreata at e12.5 when compared to wild type. Genes were identified using PancChip 6.1 microarray analysis. Statistical significance of 20% FDR and a fold change of 1.5 were used as cutoffs for determining expression changes. Genes were categorized based on function determined by gene ontology (GO) term associations.

**Table 2:** Upregulated genes in Nkx2.2^-/- ^versus Nkx2.2^+/+ ^pancreata at e12.5 and e13.5.

Genbank Accession Number	*Gene Symbol*	Gene Name	e12.5 Fold Change
**Secreted Factors**			
NM_021488	*Ghrl*	GHRELIN	6.5
NM_010491	*Iapp*	ISLET AMYLOID POLYPEPTIDE	5.2
NM_025684	*Nepn*	RIKEN CDNA 5730521E12 GENE	3.1
			
**Transmembrane Proteins**			
NM_145539	*Tm4sf4*	TRANSMEMBRANE 4 SUPERFAMILY MEMBER 4	4.5
NM_021286	*Sez6*	SEIZURE RELATED GENE 6	2.4
NM_030701	*Gpr109a*	G PROTEIN-COUPLED RECEPTOR 109A	1.9
NM_008150	*Gpc4*	GLYPICAN 4	1.8
NM_007417	*Adra2a*	ADRENERGIC RECEPTOR, ALPHA 2A	1.7
			
**Peptidases**			
NM_033612	*Ela1*	ELASTASE 1, PANCREATIC	1.9
			
**Metabolism**			
NM_011107	*Pla2g1b*	PHOSPHOLIPASE A2, GROUP IB, PANCREAS	2.7
NM_007981	*Acsl1*	ACYL-COA SYNTHETASE LONG-CHAIN FAMILY MEMBER 1	2.0
NM_007482	*Arg1*	ARGINASE 1, LIVER	1.8
			
**Chromatin**			
NM_011417	*Smarca4*	SWI/SNF RELATED, MATRIX ASSOCIATED, ACTIN DEPENDENT REGULATOR OF CHROMATIN, SUBFAMILY A, MEMBER 4	3.6
			
**Other**			
CV677510	*IMAGE = 6437263*	Unknown	1.8
NM_145616	*Lrrc49*	LEUCINE RICH REPEAT CONTAINING 49	1.7
NM_173405	*6530401C20Rik*	RIKEN CDNA 6530401C20 GENE	1.7

16 genes were upregulated in Nkx2.2^-/- ^pancreata at e12.5 and e13.5. Genes were identified using PancChip 6.1 microarray analysis and a fold change of 1.5 was used as a cutoff for determining expression changes. Genes were categorized based on function determined by gene ontology (GO) term associations.

**Table 3:** Downregulated genes in Nkx2.2^-/- ^versus Nkx2.2^+/+ ^pancreata at e12.5.

Genbank Accession Number	*Gene Symbol*	Gene Name	e13.5 Fold Change
**Secreted Factors**			
NM_008387	*Ins2*	INSULIN II	-3.4
NM_170593	*Disp2*	DISPATCHED HOMOLOG 2 (DROSOPHILA)	-1.8
NM_009523	*Wnt4*	WINGLESS-RELATED MMTV INTEGRATION SITE 4	-1.6
			
**Transcription Factors**			
NM_008393	*Irx3*	IROQUOIS RELATED HOMEOBOX 3 (DROSOPHILA)	-2.6
NM_008665	*Myt1*	MYELIN TRANSCRIPTION FACTOR 1	-2.4
NM_007960	*Etv1*	ETS VARIANT GENE 1	-1.8
NM_021459	*Isl1*	ISLET-1 TRANSCRIPTION FACTOR, LIM/HOMEODOMAIN	-1.5
			
**Transmembrane Proteins**			
NM_001014761	*Scn2b*	SODIUM CHANNEL, VOLTAGE-GATED, TYPE II, BETA	-2.0
NM_024226	*Rtn4*	RETICULON 4	-1.9
			
**Transporters**			
NM_007514	*Slc7a2*	SOLUTE CARRIER FAMILY 7 (CATIONIC AMINO ACID TRANSPORTER, Y+ SYSTEM), MEMBER 2	-2.1
NM_028924	*Mtac2d1*	MEMBRANE TARGETING (TANDEM) C2 DOMAIN CONTAINING 1	-1.6
			
**Signaling**			
NM_007832	*Dck*	DEOXYCYTIDINE KINASE	-2.3
			
**Metabolism**			
NM_007748	*Cox6a1*	CYTOCHROME C OXIDASE, SUBUNIT VI A, POLYPEPTIDE 1	-2.0
			
**Chromatin**			
NM_175074	*Hmgn3*	HIGH MOBILITY GROUP NUCLEOSOMAL BINDING DOMAIN 3	-1.7
			
**Other**			
NM_152800	*Tor2a*	TORSIN FAMILY 2, MEMBER A	-2.4

15 additional genes were downregulated in Nkx2.2^-/- ^pancreata at e13.5. Genes were identified using PancChip 6.1 microarray analysis and a fold change of 1.5 was used as a cutoff for determining expression changes. Genes were categorized based on function determined by gene ontology (GO) term associations.

### Altered expression of genes encoding transcription factors

Interestingly, a relatively large proportion (20%) of downregulated genes in the Nkx2.2^-/- ^pancreas are transcription factors (Table 1, 3, Figure 1A), whereas no transcription factors were found to be significantly upregulated (Table 2). Six of the nine transcription factors were confirmed by quantitative rt-PCR (qRTPCR) (Figure 1C); expression of irx3, hes7 and irf6 did not appear to be changed in the Nkx2.2^-/- ^pancreas at e12.5, e15.5, or e18.5 (Figure 1C and data not shown). Of the genes with altered expression, it appears that several encode transcription factors that are critical during early islet cell fate specification, including Ngn3, Myt1, NeuroD1, and MafB.

**Figure 2:**
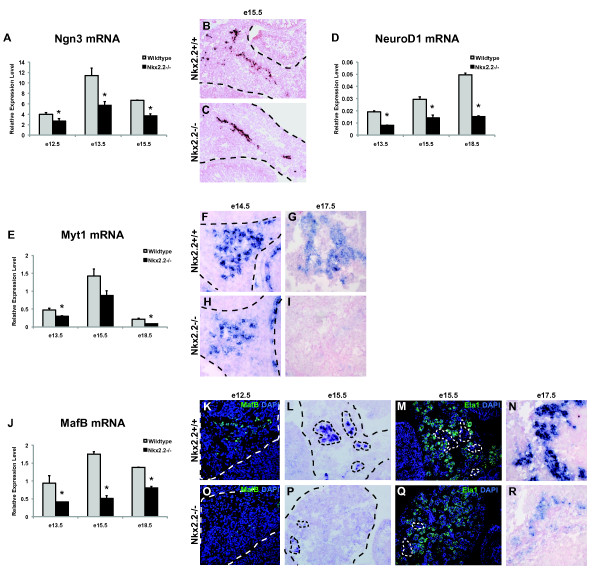
**Loss of Nkx2.2 affects expression of critical early endocrine progenitor transcription factors; Ngn3, NeuroD1, Myt1, and MafB**. qRTPCR of (A) Ngn3 levels were assessed at e12.5, e13.5, and e15.5; (D) NeuroD1, (E) Myt1, and (J) MafB transcript levels were determined at e13.5, e15.5, and e18.5 and all genes compared wild type (grey bars) and Nkx2.2^-/- ^pancreata (black bars). *In situ *hybridization comparing wild type and Nkx2.2^-/- ^pancreas at e15.5 of Ngn3 (B and C), at e14.5 and e17.5 of Myt1 (F, G, H, and I), at e15.5 and e17.5 of MafB (L, N, P, and R). Immunofluorescence staining of MafB (green) was compared between wild type (K) and Nkx2.2 null (O) pancreata at e12.5. Immunofluorescence staining of Ela1 (green) on adjacent sections at e15.5 shows restriction to the endocrine compartment (M and Q). DAPI (blue) represents nuclei. Error bars represent SEM and asterisks denote statistical significance < 0.05. Magnification 20× for all except N and R, which are 40×. Large dashed lines outline the pancreas at e12.5, e14.5 and e15.5. Small dashed lines outline MafB expression.

Ngn3 is a basic helix-loop-helix transcription factor that is essential for the formation of the endocrine islet cell population [10]. Ngn3 expression defines the endocrine progenitor cell and several knockout and lineage tracing studies have determined that Ngn3 is required for the specification of all five endocrine cell types [10,11,31]. Nkx2.2 and Ngn3 are co-expressed in the endocrine progenitor cell and in the absence of Ngn3, Nkx2.2 expression in these cells is lost [5]. In the absence of Nkx2.2, Ngn3^+ ^cell numbers remain unchanged (Figure 2B and 2C; [12]), but there appears to be a significant and sustained reduction of Ngn3 expression (Figure 2A). These combined results suggest that Ngn3 and Nkx2.2 function in a co-regulatory loop, and maintenance of their respective expression levels within the progenitor cell may be necessary to specify appropriate islet cell fates. Not surprisingly, Myelin transcription factor 1 (Myt1), a downstream target of Ngn3 [40], is significantly reduced in the Nkx2.2^-/- ^mice. Myt1 has also been shown to be important for appropriate endocrine pancreas differentiation and, at e13.5, over 60% of Ngn3+ cells also express Myt1 [41]. Furthermore, Myt1 and Ngn3 are believed to form a feed-forward loop to promote endocrine differentiation [40]. A significant reduction of Myt1 expression in the Nkx2.2^-/- ^mice is not apparent until e13.5, which may suggest that its down-regulation is secondary to the loss of Ngn3. qRTPCR analysis confirmed that Myt1 expression becomes decreased at e13.5 and the reduction in expression is maintained throughout gestation (Figure 2E). RNA *in situ *hybridization analysis at e14.5 and e17.5 further verified decreased Myt1 expression throughout the pancreatic epithelium (Figure 2F, G, H, and 2I). In summary, our data suggest that Nkx2.2 functions within the Myt1 and Ngn3 regulatory loop. Future studies will elaborate the precise regulatory connections between these three factors to specify appropriate islet cell fate decisions.

NeuroD1 is a basic helix-loop-helix transcription factor that has also been shown to function directly downstream of Ngn3. Consistent with the reduction of Ngn3 expression, NeuroD1 is downregulated in the Nkx2.2^-/- ^mice (Figure 2D; [42]). Furthermore, we have recently determined that Nkx2.2 and Ngn3 cooperate to directly activate the NeuroD1 promoter [43], suggesting that the reduction of NeuroD1 expression could be caused by both direct and indirect regulation. Previous experiments also indicate that Nkx2.2 and NeuroD1 genetically interact in the alpha cell population [42]. It is likely that these complex regulatory and functional interactions between Nkx2.2, Ngn3, Myt1 and NeuroD1 are necessary to precisely control early islet cell fate decisions.

We also detected a significant reduction in MafB expression. Two members of the large Maf transcription factor family, MafA and MafB, are important for endocrine pancreas development [44-49]. MafA, a mature β cell marker, is a direct transcriptional target of Nkx2.2 and its expression is absent in Nkx2.2^-/- ^mice [38]. In this study, the comparative gene expression analysis was performed at a time point prior to MafA expression in the pancreas (MafA signal was undetectable in both wild type and mutant pancreatic e12.5 and e13.5 samples) and therefore MafA was not included on our list of downregulated genes. MafB is expressed prior to MafA and is critical for appropriate differentiation of α and β cells [46,50]. Here we demonstrate that MafB expression is decreased in Nkx2.2^-/- ^mice at e12.5 (Table 1). Further verification by qRTPCR confirmed that decreased expression of MafB was maintained throughout gestation, with the largest observed differences occurring at e15.5 (Figure 2J, L, and 2P). Although it is possible that the reduction in MafB expression is due to the loss of α and β cells, as shown by a reduction of MafB^+ ^cells at e12.5 (Figure 2K and 2O), MafB *in situ *hybridization analysis suggests that MafB expression is also reduced on a per cell basis throughout the pancreatic epithelium (Figure 2N and 2R). We have also determined that Nkx2.2 binds directly to a highly conserved DNA sequence within the MafB 5' upstream regulatory region (Torres and Sussel, unpublished data), suggesting that Nkx2.2 may regulate both MafA and MafB to impact the early specification of α and β cells in addition to their proper maturation state.

Isl1 and Etv1 (ER81) show differential expression by e13.5 in the Nkx2.2^-/- ^mice (Table 3). Isl1 is necessary for dorsal pancreatic mesenchyme and endocrine cell differentiation [51]. Etv1, an Ets domain family member, is expressed in the pancreas [52,53]; however, its function during pancreas organogenesis remains unknown. Etv1 is important in neuronal differentiation and neurogenesis [54,55] and has recently been shown to form a transcriptional complex with Sox9, a characterized pancreatic progenitor marker, in breast cancer cells [56-58]. Interestingly, Isl1, and Etv1 appear to be shared downstream (indirect or direct) targets of Nkx2.2 and Ngn3 (Additional File 2: Table S1). The complex regulatory relationship between Nkx2.2 and Ngn3 suggests that these factors function in the same pathway or in parallel to regulate Isl1 and Etv1. Further elucidation of the co-regulatory pathways and intricate feedback mechanisms that we have uncovered in these studies will facilitate our understanding of elaborate transcriptional networks that regulate islet cell fate decisions.

### Altered expression of genes encoding transmembrane and secreted factors

Two particularly interesting classes of genes that displayed altered regulation in the Nkx2.2^-/- ^pancreas are transmembrane proteins (9.3% downregulated and 31% upregulated; Figure 1A and 1B) and secreted factors (17% downregulated and 19% upregulated; Figure 1A and 1B). These classes of proteins are particularly interesting because they have the potential to interface with environmental and extracellular signals to direct cellular function. To determine whether these gene alterations were due to the loss or gain of specific cell types, or due to aberrant ectopic expression, we analyzed gene expression changes over the course of embryonic development and performed mRNA *in situ *analysis to define their spatial localization within the pancreas.

**Figure 3:**
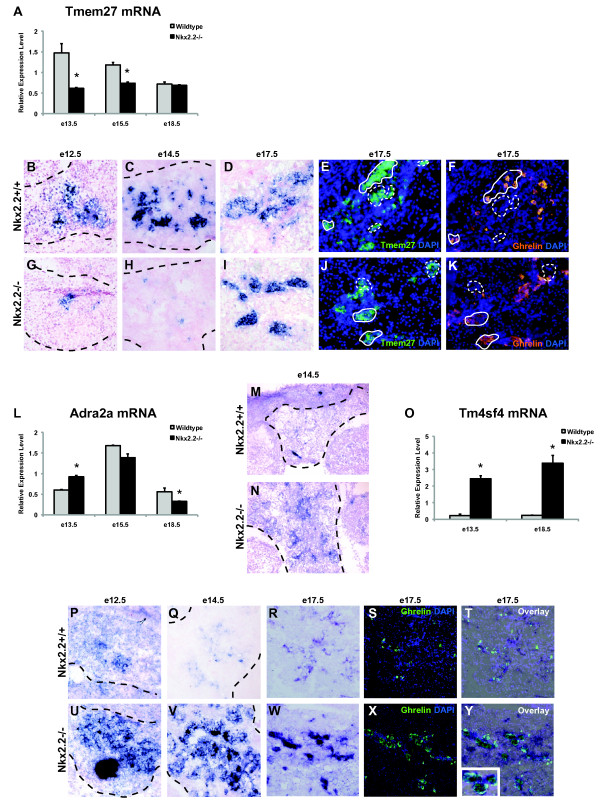
**Loss of Nkx2.2 affects expression of known endocrine transmembrane proteins, Tmem27, Adra2a, and a novel tetraspanin, Tm4sf4**. qRTPCR of (A) Tmem27, (L) Adra2a, and (O) Tm4sf4 mRNA transcript levels were determined at e13.5, e15.5, and e18.5 and all genes compared wild type (grey bars) and Nkx2.2^-/- ^pancreata (black bars). *In situ *hybridization comparing wild type and Nkx2.2^-/- ^pancreas at e12.5, e14.5, and e17.5 of Tmem27 (B, C, D, G, H, and I), e14.5 of Adra2a (M and N), and e12.5, e14.5, and e17.5 of Tm4sf4 (P, Q, R, U, V, and W). Error bars represent SEM and asterisks denote statistical significance < 0.05. To assess co-expression, immunofluoresence staining of Tmem27 (E and J;green) and Ghrelin (F and K;copper) was performed on adjacent sections. Similarly, adjacent sections to the Tm4sf4 *in situ *hybridizations were stained for ghrelin (T and Y;green) and overlayed (T and Y: inset 2× zoom). DAPI (blue) indicates nuclei. Magnification 20× for e12.5, e14.5, R, S, T, W, X, Y, while 40× for E, F, J, and K. Large black dashed lines outline the pancreas at e12.5 and e14.5. Small solid white lines outline Tmem27 and ghrelin co-expression areas. Dashed white lines outline Tmem27+ cells that do not co-express ghrelin.

The transmembrane protein Tmem27, also known as collectrin, is expressed in multiple endocrine cell types during development, including α and β cells, but becomes restricted to β cells in the adult where it functions to increase β cell proliferation *in vivo *[59]. Tmem27 expression is increased in hypertrophied islets and has been shown to function in β cell lines and primary β cells to enhance glucose stimulated insulin secretion [60]. However, *in vivo *loss of Tmem27 did not affect overall islet number, β cell mass, or function; islet composition was not assessed in this study [61]. As would be expected of a factor that is expressed in α and β cells during development, Tmem27 is significantly reduced in e12.5 and e13.5 Nkx2.2^-/- ^mice, which have less α cells and completely lack β cells (Table 1). Unexpectedly, however, by late gestation Tmem27 transcript is expressed at equivalent levels throughout the endocrine compartment in wild type and Nkx2.2^-/- ^pancreas, as measured both by qRTPCR (Figure 3A) and RNA *in situ *hybridization analysis (Figure 3B, C, and 3D and 3G, H, and 3I). Furthermore, immunofluorescence staining of Tmem27 and ghrelin on adjacent sections of e17.5 wild type and Nkx2.2^-/- ^pancreas (Figure 3E, F, J, and 3K) confirm that some ghrelin cells normally co-express Tmem27 and this expression is maintained in the Nkx2.2^-/- ^ghrelin-expressing population. Therefore, the early reduction in Tmem27 expression in the Nkx2.2^-/- ^pancreas could be attributed to the absence of the α and β cell populations, and the restoration of normal Tmem27 expression levels could be due to its expression in the ghrelin cell population that has now replaced the missing islet cell types. However, this does not account for the loss of Tmem27 expression during midgestation (e13.5 e15.5) when the replacement ghrelin cells have already formed. This would suggest that there is a transient reduction or loss of Tmem27 expression in mutant pancreas during the secondary transition that may contribute to, or be caused by, the cell fate switch that is occurring in the Nkx2.2^-/- ^pancreas.

In contrast to Tmem27, Adra2a, a G-protein coupled receptor, has increased expression early in Nkx2.2^-/- ^pancreas development, but becomes decreased in late Nkx2.2^-/- ^pancreas (Table 2, Figure 3L). Adra2a is normally expressed in pancreatic islets [62,63] where it functions as a β cell postsynaptic receptor to inhibit insulin secretion [64,65]. The loss of Adra2a expression late in gestation is consistent with the absence of β cells. However, the elevated expression of Adra2a in the Nkx2.2^-/- ^pancreas between e12.5 - e14.5 (Table 2, Figure 3L, M, and 3N) suggests that Adra2a might also be expressed in non-β cell populations prior to β cell formation. The early Adra2a gene expression changes that occur in response to loss of Nkx2.2 activity may be indicative of a functional role for Adra2a outside of the β cell.

One of the most highly upregulated genes identified in the Nkx2.2^-/- ^pancreas by the microarray analysis is Tm4sf4, a member of the L6 domain superfamily of tetraspanin surface proteins [66]. Tm4sf4 is expressed in non-dividing hepatocytes [67] and the human orthologue, il-TMP, is found in non-dividing intestinal epithelial cells [68]. Expression of Tm4sf4 in the pancreas has not been previously reported. Our analysis shows that in wild type pancreas, Tm4sf4 is normally expressed at low levels throughout the pancreatic epithelium at all stages examined (Figure 3O, P, Q, and 3R). Furthermore, Tm4sf4 is enriched in FACs-purified Ngn3-EGFP cells (Balderes and Sussel, unpublished data; [13]) and is upregulated in pancreata that have undergone exocrine to endocrine reprogramming [69]. In the Nkx2.2^-/- ^pancreas, qRTPCR and mRNA *in situ *analysis demonstrated that Tm4sf4 expression is increased in the pancreas throughout gestation (Table 2, Figure 3O, P, Q, and 3R and 3U, V, and 3W). At early stages of pancreas specification, Tm4sf4 appears to be broadly expressed throughout the Nkx2.2^-/- ^pancreatic epithelium (Figure 3P, Q, U, and 3V), but beginning at e15.5, Tm4sf4 mRNA becomes restricted to the ductal and endocrine compartment in the Nkx2.2^-/- ^pancreata (Figure 3R, W, and data not shown). Interestingly, at e14.5 we observed a subpopulation of Tm4sf4^+ ^cells in Nkx2.2^-/- ^pancreata that appear to express extremely high levels of Tm4sf4 within the ductal epithelium (Figure 3Q and 3V). Antibodies against Tm4sf4 are not available to precisely determine cell type localization; however, analysis of Tm4sf4 mRNA and ghrelin protein expression on adjacent sections demonstrates that Tm4sf4 is restricted to the ductal and endocrine compartments, and partially co-localizes with the ghrelin population in the e17.5 Nkx2.2^-/- ^pancreas (Figure 3R, S, W, X, and 3Y). Future studies will focus on pancreatic cell type expression and subcellular localization of Tm4sf4. Closely-related Tm4sf4 family members and other tetraspanins are known to interact with integrins, and are often involved in EMT, cell adhesion mechanisms and/or migration events [70-73]. Since Tm4sf4 appears to be enriched in the Ngn3+ cell population and is one of the most highly upregulated genes in the Nkx2.2^-/- ^pancreata, we speculate that the aberrant upregulation of Tm4sf4 in the endocrine progenitor cells of the Nkx2.2^-/- ^mice may interfere with normal cell-cell interactions and/or the interpretation of extrinsic signals to prevent appropriate islet cell differentiation. Furthermore, the Tm4sf4 gene contains two highly conserved Nkx2.2 binding sites (data not shown), suggesting that Tm4sf4 might function directly downstream of Nkx2.2 in the progenitor population to modulate islet cell fate choice.

### Altered expression of exocrine genes

Surprisingly, although we had not previously detected a change in exocrine cell numbers, several genes expressed in the exocrine compartment also display altered expression in the Nkx2.2^-/- ^pancreas. The Nkx2.2^-/- ^mice do not display overt exocrine pancreatic defects [19,34]; however, Nkx2.2 is initially co-expressed with Pdx1 and Ptf1a in the pancreatic progenitors that give rise to all the pancreatic cell types. It is therefore possible that Nkx2.2 plays a transient role in exocrine gene regulation. Whether these gene expression changes translate into functional defects cannot be determined in the Nkx2.2^-/- ^animals, which die shortly after birth.

**Figure 4:**
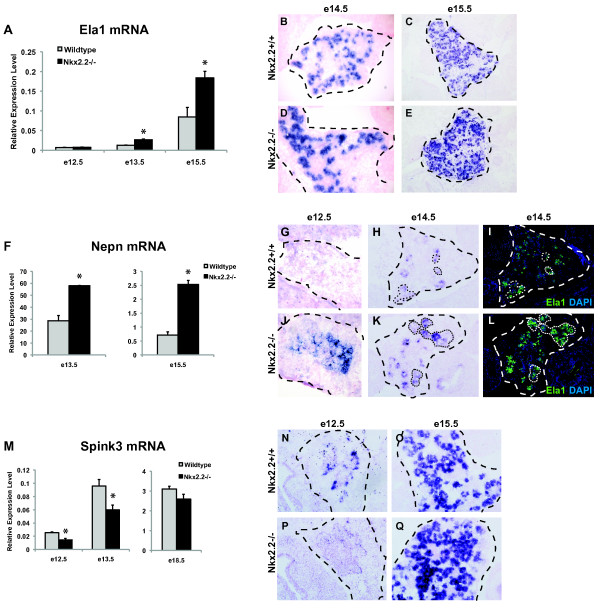
**Loss of Nkx2.2 affects expression of known exocrine factors, Ela1, Spink3, and a novel secreted proteoglycan, Nepn**. qRTPCR of (A) Ela1 mRNA was assessed at e12.5, e13.5, and e15.5, (F) Nepn mRNA was assessed at e13.5 and e15.5, and (M) Spink3 mRNA transcript levels were determined at e12.5 and e13.5, while all gene expression analyses compared wild type (grey bars) and Nkx2.2^-/- ^pancreata (black bars). *In situ *hybridization comparing wild type and Nkx2.2^-/- ^pancreas at e14.5 and e15.5 for Ela1 (B, C, D, and E), e12.5 and e14.5 for Nepn (G, H, J, and K), e12.5 and e15.5 for Spink3 (N, O, P, and Q). To show co-expression of Nepn in the exocrine compartment and confirm increased protein levels, Ela1 immunofluorescence was performed on adjacent sections (I and L;green). DAPI (C and E; blue) indicates nuclei. Error bars represent SEM and asterisks denote statistical significance < 0.05. Magnification 20×. Large dashed lines outline the pancreas at e12.5, e14.5, and e15.5. Small dashed lines outline areas of Nepn and Ela1 co-expression.

Elastase 1 (Ela1) is a digestive enzyme produced within the pancreatic acini [74,75]. In the Nkx2.2^-/- ^pancreata, Ela1 appears to be upregulated as early as e13.5 when both exocrine and endocrine cell types are differentiating, but prior to acini maturation (Table 2). We confirmed elevated mRNA expression at e13.5 and e15.5 by qRTPCR (Figure 4A) and demonstrated that the elevated Ela1 was restricted to the exocrine compartment at e14.5 and e15.5 using mRNA *in situ *hybridization and immunofluorescence analysis (Figure 4B-E, I and 4L). It therefore appears that there may be an increase in elastase expression per cell, rather than ectopic expression of elastase.

Nephrocan (Nepn), a secreted glycoprotein of the kidney that can inhibit TGFβ signaling and is important for a variety of essential developmental events including epithelial to mesenchymal transitions (EMT) and cell migration [76], also displays increased expression in the Nkx2.2^-/- ^pancreas at e12.5, e13.5, e14.5, and e15.5 (Table 2, Figure 4F, G, H, J, and 4K). Nepn has not been previously reported in the pancreas, however, in both wild type and Nkx2.2^-/- ^pancreas, Nepn expression is detectable in the exocrine compartment and overlaps with the elastase expression domain (Figure 4H, I, K, and 4L). In the embryonic kidney, Nepn is expressed transiently, with its highest expression levels peaking at e11.5 [76]. Similarly, in the Nkx2.2^-/- ^pancreas, we could detect a large increase in Nepn expression between e13.5 and e15.5, but by e18.5 Nepn expression could not be detected by either qRTPCR or mRNA *in situ *analysis (Figure 4F, G, H, J, K, and data not shown). Furthermore, in both wild type and Nkx2.2^-/- ^pancreas Nepn mRNA expression was already considerably lower at e15.5 when compared to e13.5, although even at e15.5, Nepn levels were still significantly higher in the mutant pancreas compared to wild type (Figure 4F; note y-axis scale). This would suggest that Nepn is transiently upregulated in the exocrine pancreas of the Nkx2.2^-/- ^mice.

The exocrine gene serine protease inhibitor Kazal type 3 (Spink3) [77-79] is also affected in Nkx2.2^-/- ^pancreata, but unlike Ela1 and Nepn, Spink3 is downregulated in the Nkx2.2^-/- ^pancreas at e12.5 (Table 1). In wild type pancreas, Spink3 mRNA can be detected as early as e11.5 and is expressed in acini by e13.5 continuing through e18.5. Spink3 is also expressed in the intestine, kidney, and some reproductive tissues during embryogenesis [77]. Recent functional analysis suggests that Spink3 has essential roles in the maintenance of the integrity and regeneration of acinar cells. qRTPCR and *in situ *hybridization of Spink3 mRNA levels in the Nkx2.2^-/- ^pancreas confirmed the downregulation of Spink3 at e12.5 (Figure 4M, N, and 4P) and e13.5 (Figure 4M). Spink3 mRNA levels in wild type and Nkx2.2 null pancreata appear to equilibrate between e15.5 and e18.5 (Figure 4M, O, and 4Q), suggesting that the upregulation of Spink3 is a transient event.

A number of additional known exocrine genes are also downregulated in the Nkx2.2^-/- ^mice at e12.5, including pancreatic lipase-related protein, chymotrypsinogen B1 and carboxypeptidase 2 (Table 1). There are no overt changes in the number of exocrine cells or their morphology and polarity in the Nkx2.2^-/- ^mice to suggest a defect in the formation of the exocrine cell population [19]. Furthermore, Nkx2.2 expression normally becomes extinguished in the differentiated acini cells. We predict that the observed exocrine gene expression changes reflect differences in the regulation of these genes in the pancreatic progenitor population. It is possible that these gene expression changes are transient (as in the case of Nepn and Spink3) or, alternatively, they may initiate events that lead to persistent functional changes in the acini that are as yet uncharacterized.

## Conclusion

**Figure 5:**
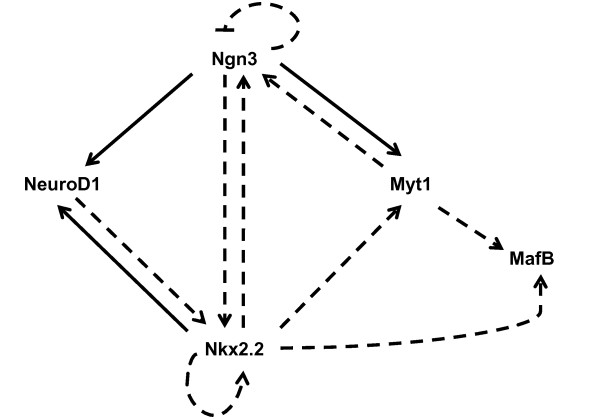
**A proposed model for an Nkx2.2 role during endocrine cell specification at the secondary transition**. The solid arrows indicate direct activation that is based on published regulatory data, while dashed arrows represent untested direct regulatory interactions proposed here as well as by other groups and in some cases known indirect regulation.

Although the Nkx2.2^-/- ^mice display a severe islet cell misspecification phenotype, our microarray studies identified very few altered transcription factor changes that contribute to these changes during the secondary transition. What the global gene expression profiling suggests, however, is that Nkx2.2 plays a predominant role in regulating islet cell fate choices within the Ngn3 regulatory circuit. Nkx2.2 is not likely to function upstream of this regulatory junction, since the loss of Nkx2.2 does not affect the expression of Pdx1, Ptfl1a or Sox9 (Figure 1 and data not shown). We do, however, demonstrate that Ngn3 and its functional partners, Myt1 and NeuroD1, all have reduced expression in Nkx2.2^-/- ^mice. The reduction of Myt1 and NeuroD1 may be secondary to the reduction of Ngn3; however, there are several lines of evidence to suggest that the regulatory and functional inter-relationships between these proteins are more complex, as summarized in Figure 5. It has been demonstrated that Nkx2.2 expression is reduced in Ngn3^-/- ^mice indicating that these proteins function in a feedback loop [5]. Furthermore, Myt1 expression is decreased in Ngn3^-/- ^mice, but Myt1 has also been shown to induce Ngn3 activation, and Myt1 and Ngn3 apparently cooperate in their regulation of islet differentiation [40,41]. Additional studies have demonstrated that ectopic expression of NeuroD1 activates Nkx2.2 and it has been suggested that Ngn3 and Nkx2.2 autoregulate their own transcription [80]. These complex regulatory interactions are likely necessary to fine tune this critical juncture in islet cell specification. Precise regulation of the expression levels and activity of each of these essential factors may be necessary for the appropriate initiation of the critical developmental events that function downstream of the Ngn3 progenitor, including the regulation and co-regulation of downstream targets, such as MafB, Isl1 and Etv1. Rather than the simple linear regulatory pathway that is often invoked, an intricate web of feedback and feedforward regulation must be necessary to induce appropriate islet cell specification.

In this study, we have identified several known and novel secreted and transmembrane proteins that may function downstream of Nkx2.2 to mediate differentiation events. Transmembrane proteins such as Tm4sf4 or signaling molecules such as nephrocan may function in or on the endocrine progenitor cells to translate the cell's intrinsic signals to the environment or vice versa. The identification and characterization of transmembrane and secreted molecules that influence islet cell formation will provide critical information about the extrinsic signals that are necessary to manipulate pancreatic development and cell differentiation. In support of this, a recent study demonstrated the tetraspanin Tm4sf3 mediates fusion of the dorsal and ventral buds, as well as acinar cell differentiation during *Xenopus *pancreatic development [81].

Finally, these studies demonstrate for the first time that Nkx2.2 indirectly or directly regulates the expression of several exocrine genes. The functional impact caused by misregulation of these exocrine genes is not clear; however, it suggests that Nkx2.2 also plays a minor role in non-endocrine lineages. It is possible that the regulation of exocrine gene expression occurs in the "Nkx2.2 low" expressing cells that are present in the Pdx1^+^, Ngn3^- ^pancreatic progenitors [5]. This is similar to the finding that deletion of Nkx6.1, a β cell transcription factor that is co-expressed with Nkx2.2 in the early pancreatic epithelium, transiently upregulates the expression of the exocrine factor Nkx6.2 in Pdx1^+ ^pancreatic progenitor cells [82]. Misregulation of additional exocrine genes was not reported in the Henseleit study. However, we have determined that Nkx6.2 is also upregulated in the Nkx2.2^-/- ^pancreas (data not shown; note: Nkx6.2 was not present on PancChip6.1).

In summary, the results of our comparative expression analysis of Nkx2.2^-/- ^and wild type pancreata begins to illustrate the cell type specific requirements for Nkx2.2 in the regulation of endocrine and exocrine gene expression and will ultimately help to dissect out the molecular functions of Nkx2.2 in regulating important pancreatic cell fate decisions.

## Methods

### Mice

Nkx2.2^+/- ^heterozygous mice were previously generated by homologous recombination [19] and were maintained on a Swiss Black (Taconic) background. Genotyping of mice and embryos was performed by PCR analysis as previously described [19,42]. Mice were housed and treated according to Columbia University and UCDHSC IACUC approval protocols.

### RNA isolation

Nkx2.2^+/- ^heterozygous males and females were mated and plugged in order to know specific embryonic stage. An n = 5 for e12.5 and e13.5 pancreata was collected for each experimental group (wt and Nkx2.2^-/-^). Pancreata were dissected from embryos and stored in RNAlater (Ambion/ABI, TX) at 4°C until genotyping could be completed. Once genotyped, two pancreata were pooled for each e12.5 experimental group and the following numbers of wild type and Nkx2.2^-/- ^pancreata were pooled for e13.5 experimental groups: n1 = 5wt, 6ko; n2 = 4wt, 5ko; n3 = 4wt, 7ko; n4 = 4wt, 6ko; n5 = 3wt, 3ko. For each pooled set, RNA was isolated using an RNeasy Micro Kit (Qiagen, CA) without the DNase incubation step. The concentration of the RNA samples was determined using the NanoDrop^® ^ND-1000 UV-Vis Spectrophotometer. RNA samples were analyzed using an Agilent 2100 Bioanalyzer Lab-On-A-Chip Agilent 6000 Series II chip to determine the integrity of the samples. The RNA was of high quality with an RNA integrity number > 8.

### RNA Amplification, Labeling, and Hybridization

For e12.5 RNA, approximately 200 ng of total RNA was amplified using the MessageAmpTM II aRNA Amplification Kit (Ambion/ABI, TX). After amplification 2.5 μg of aRNA was indirectly labeled using amino-allyl dUTP and anchored oligo(d)T prime reverse transcription. For e13.5 RNA, approximately 50 ng of total RNA was amplified using the Ovation™ Aminoallyl RNA Amplification and Labeling System (Nugen Inc, CA). The amplification process resulted in a yield of 6-10 μg of amplified cDNA, which was purified using the QIAquick PCR Purification Kit (Qiagen, CA) and then eluted in coupling buffer (0.1 M Sodium Bicarbonate, pH 9). 2 μg of each cDNA was coupled with the appropriate Cy3 or Cy5 fluorescent label (Cy™ Dye, Amersham Pharmacia Biotech Ltd, NJ), combined and purified using the MinElute PCR Purification Kit (Qiagen, CA). After coupling, the Cy3 and Cy5 samples were combined and purified using the MinElute PCR Purification Kit (Qiagen, CA). After purification, 2.5 μg of Mouse Cot1 DNA (Invitrogen Life Technologies) and 2.5 μg Oligo-dT were added to each sample and denatured at 95°C for 5 min. The samples were then cooled to 42°C and an equal volume of 2× hybridization buffer (50% formamide, 10× SSC, and 0.2% SDS) was added, mixed, and applied to the Mouse PancChip 6.1 cDNA microarray slide.

### Microarray

The Mouse PancChip 6 contains 13,059 mouse cDNAs chosen for their expression in various stages of pancreatic development, many of which are not found on commercially available arrays [83]. Stringent quality control measures resulted in the sequence verification of all probes on the array. The results are presented as downloadable gene lists, available at: https://www.cbil.upenn.edu/RADQuerier/php/displayStudy.php?study_id=3061

### Scanning and Image Analysis

Microarray slides were hybridized overnight, then washed and scanned with an Agilent G2565BA Microarray Scanner. Images were analyzed with GenePix 5.0 software (Axon Instruments). Median foreground intensities were obtained for each spot and imported into the mathematical software package "R", which was used for all data input, diagnostic plots, normalization and quality checking steps of the analysis process using scripts developed in-house by PW. The ratio of expression for each element on the array was calculated in terms of M (log2(Red/Green)) and A ((log2(Red) + log2(Green))/2)). The dataset was filtered to remove positive control elements (Cy3 anchors and SpotReport elements) and any elements that had been manually flagged as bad. The M values were then normalized by the print tip loess method using the "marray" microarray processing package in "R". Statistical analysis was performed in "R" using both the LIMMA and SAM packages. For the e12.5 study, 4 out of the 5 hybridizations passed quality control specifications and were subsequently used for analysis. For the e13.5 study all 5 hybridizations were analyzed.

### Quantitative Real-Time PCR

Total RNA was harvested from e12.5, e13.5, e15.5, and e18.5 whole pancreata using the RNeasy Micro or Mini Kit (Qiagen). Experimental n = 3 for each embryonic age. For each age group 1 μg total RNA was converted to cDNA using the Superscript III Kit (Invitrogen). qRTPCR was performed using custom primers and SYBR green (Applied Biosystems) or Taqman (Applied Biosystems) primer and probe sets. Refer to Additional File 3: Table S2 for all primer/probe sets used. Gene of interest relative mRNA expression levels were determined by using the standard curve method (User Bulletin #2, ABI Prism 7700 Sequence Detection System, Applied Biosystems) for each sample and was subsequently normalized to relative Cyclophilin B mRNA levels. All qRTPCR single-plex reactions were performed on an ABI PRISM 7000 Sequence Detection System.

### *In situ *hybridization

RNA *in situ *hybridization was performed as previously described [34] on e12.5, e14.5, and e17.5 whole embryo frozen 8 μm sections that were fixed overnight with 4% paraformaldehyde. The following cDNA clones were ordered from Open Biosystems: Adra2a (EMM1002-22629), Ela1 (MMM1013-65444), MafB (MMM1013-7514203), Myt1 (MMM1013-9334659), Spink3 (EMM1002-6296766), Tmem27 (MMM1013-9200171), Tm4sf4 (MMM1013-65619). Nepn cDNA was cloned from isolated kidney total RNA with the following primers: forward 5'-GCAATGCACCCGCTTTGGGCTTTTC, reverse 5'-ATCTATTTCATAATCGTCATCGTCGTC. The cDNA clones encoding full-length Nkx2.2 and Ngn3 were described previously [19,84]. cDNA plasmids were linearized and *in situ *antisense probes were transcribed with the following restriction enzymes and polymerases: Adra2a (EcoRI, T7), Ela1 (EcoRI, T7), MafB (EcoRI, T7), Myt1 (SalI, T3), Nepn (XhoI, Sp6), Ngn3 (BamHI, T7), Nkx2.2 (NotI, T7), Spink3 (EcoRI, Sp6), Tmem27 (EcoRI, T7), and Tm4sf4 (SmaI, T7). Sense probes were also tested for each gene without any evidence of non-specific staining. *In situ *hybridizations were performed on wild type and Nkx2.2^-/- ^littermate embryos. Pictures were acquired on a Leica CTR 5000 with 20× magnification.

### Immunofluorescence

Immunofluorescence was performed on e12.5, e14.5, and e17.5 whole embryo frozen 8 μm sections that were fixed overnight with 4% paraformaldehyde. Rabbit α-Elastase1 antibody (Abcam, ab21593) was used at 1:1000. Rabbit α-ghrelin (Phoenix Pharmaceuticals, CA) was used at 1:200. Rabbit α-Tmem27 (M. Stoffel, ETH Zurich) was used at 1:200. Rabbit a-MafB (Bethyl Laboratories, TX) was used at 1:1000. Donkey a-rabbit-Cy5 or α-rabbit-Cy2 (Jackson ImmunoResearch, PA) were used at 1:300. DAPI (Invitrogen, CA) was used at 1:1000 and incubated for 30 min. Confocal images were taken on a Zeiss META LSM 510.

## Authors' contributions

KRA performed all the experimental studies described in this manuscript, except the microarray analysis, and drafted the manuscript. PW performed the microarray analysis, statistical analysis and assisted with the generation of Figure 1. KHK assisted with the study design, supervised the microarray analysis and provided important intellectual input to the project. LS conceived of the study, participated in its design and coordination and helped draft the manuscript. All authors read and approved the final manuscript.

## Supplementary Material

Additional file 1 **Figure S1 - Nkx2.2 is expressed broadly throughout early pancreatic epithelium**. *In situ *hybridization of Nkx2.2 at e13.5 in wild type pancreas. Magnification 20×. Dashed line outlines the pancreas. Click here for file

Additional file 2 **Table S1 - Genes co-regulated by Nkx2.2 and Ngn3**. Genes identified as changed from Nkx2.2^-/- ^pancreata microarrays were compared to genes altered in Ngn3^-/- ^pancreata [26] or genes increased and decreased in expression between whole pancreas and Ngn3+ cell populations [28]. For each gene, it is listed whether there was an increase or decrease in Nkx2.2^-/- ^pancreata expression from e12.5 to e13.5 and what the corresponding finding was in either of the Ngn3 studies. Click here for file

Additional file 3 **Table S2 - SYBR and Taqman primer/probe sets used for qRTPCR analysis**. Oligo sequences for gene specific primers/probes. Genes are listed in alphabetical order and all sequences displayed in the 5' to 3' orientation. Click here for file
