# Supplementary figures and images for: Identification of known and novel pancreas genes expressed downstream of Nkx2.2 during development
Source: BMC Dev Biol. 2009 Dec 10;9:65. doi: 10.1186/1471-213X-9-65 (PMC2799404; doi:10.1186/1471-213X-9-65)

## Supplemental Figure 1

*Nkx2.2 in situ*

e13.5 Wildtype

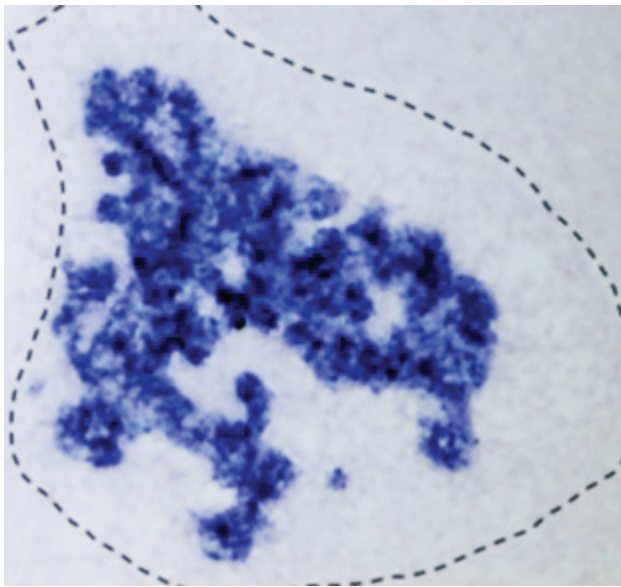

Supplement: Additional file 1 — Figure S1 - Nkx2.2 is expressed broadly throughout early pancreatic epithelium. In situ hybridization of Nkx2.2 at e13.5 in wild type pancreas. Magnification 20×. Dashed line outlines the pancreas. [file 1471-213X-9-65-S1.PDF]
