# Supplementary material for: Identification of known and novel pancreas genes expressed downstream of Nkx2.2 during development
Source: BMC Dev Biol. 2009 Dec 10;9:65. doi: 10.1186/1471-213X-9-65 (PMC2799404; doi:10.1186/1471-213X-9-65)
Supplement: Additional file 2 — Table S1 - Genes co-regulated by Nkx2.2 and Ngn3. Genes identified as changed from Nkx2.2-/- pancreata microarrays were compared to genes altered in Ngn3-/- pancreata [26] or genes increased and decreased in expression between whole pancreas and Ngn3+ cell populations [28]. For each gene, it is listed whether there was an increase or decrease in Nkx2.2-/- pancreata expression from e12.5 to e13.5 and what the corresponding finding was in either of the Ngn3 studies. [file 1471-213X-9-65-S2.PDF]

Supplemental Table 1. Gene comparison between Nkx2.2-/- data and Ngn3 microarray data sets.

Genbank Accession Number	*Gene Symbol*	Gene Name	e12.5-e13.5 Nkx2.2-/-	Comparison
**Secreted Factors**				
NM_145435	*Pyy*	PEPTIDE YY	Decreased	Downregulated at e18.5 in Ngn3-/-
NM_007694	*Chgb*	CHROMOGRANIN B	Decreased	Enriched in e13.5 Ngn3+ population<br>Downregulated at e18.5 in Ngn3-/-
NM_170593	*Disp2*	DISPATCHED HOMOLOG 2 (DROSOPHILA)	Decreased	Downregulated at e18.5 in Ngn3-/-
NM_010491	*Iapp*	ISLET AMYLOID POLYPEPTIDE	**Increased**	Downregulated at e18.5 in Ngn3-/-
**Transcription Factors**				
NM_010894	*Neurod1*	NEUROGENIC DIFFERENTIATION 1	Decreased	Enriched in e13.5 Ngn3+ population<br>Downregulated at e12.5, e15.5, e18.5 in Ngn3-/-
NM_178083	*Irf6*	INTERFERON REGULATORY FACTOR 6	Decreased	Enriched in e13.5 Ngn3+ population
NM_010658	*Mafb*	V-MAF MUSCULOAPONEUROTIC FIBROSARCOMA ONCOGENE FAMILY, PROTEIN B (AVIAN)	Decreased	Enriched in e13.5 Ngn3+ population<br>Downregulated at e18.5 in Ngn3-/-
NM_008665	*Myt1*	MYELIN TRANSCRIPTION FACTOR 1	Decreased	Enriched in e13.5 Ngn3+ population
NM_007960	*Etv1*	ETS VARIANT GENE 1	Decreased	Downregulated at e18.5 in Ngn3-/-
NM_021459	*Isl1*	ISLET-1 TRANSCRIPTION FACTOR, LIM/HOMEODOMAIN	Decreased	Enriched in e13.5 Ngn3+ population<br>Downregulated at e15.5, e18.5 in Ngn3-/-
**Transmembrane Proteins**				
NM_020626	*Tmem27*	TRANSMEMBRANE PROTEIN 27	Decreased	Downregulated at e18.5 in Ngn3-/-
NM_144926	*Sez6l2*	SEIZURE RELATED 6 HOMOLOG LIKE 2	Decreased	Downregulated at e18.5 in Ngn3-/-
**Peptidases**				
NM_008792	*Pcsk2*	PROPROTEIN CONVERTASE SUBTILISIN/KEXIN TYPE 2	Decreased	Enriched in e13.5 Ngn3+ population<br>Downregulated at e18.5 in Ngn3-/-
NM_013494	*Cpe*	CARBOXYPEPTIDASE E	Decreased	Enriched in e13.5 Ngn3+ population
**Signaling**				
NM_145736	*Pim2*	PROVIRAL INTEGRATION SITE 2	Decreased	Enriched in e13.5 Ngn3+ population
**Metabolism**				
NM_018874	*Pnliprp1*	PANCREATIC LIPASE RELATED PROTEIN 1	Decreased	Enriched in e13.5 Ngn3- population
