# Supplementary material for: Identification of known and novel pancreas genes expressed downstream of Nkx2.2 during development
Source: BMC Dev Biol. 2009 Dec 10;9:65. doi: 10.1186/1471-213X-9-65 (PMC2799404; doi:10.1186/1471-213X-9-65)
Supplement: Additional file 3 — Table S2 - SYBR and Taqman primer/probe sets used for qRTPCR analysis. Oligo sequences for gene specific primers/probes. Genes are listed in alphabetical order and all sequences displayed in the 5' to 3' orientation. [file 1471-213X-9-65-S3.PDF]

Supplemental Table 2

Gene Symbol	QPCR type	Forward Primer 5' -> 3'	Reverse Primer 5' -> 3'	Probe 5' -> 3'
**Adra2a**	SYBR	cca tca gcc ttg acc gct ac	ggt tgt act cga tgg cct gc	
**CyclophilinB**	SYBR	agg tcc tgg cat ctt gtc ca	gaa ccg ttt gtg ttt ggt cca	
**Disp2**	SYBR	caa agt cct gat ggc aca cg	gcc atg agt atc gaa gcg ga	
**Ela1**	Taqman	Applied Biosystems #Mm00712898_m1		
**Etv1**	SYBR	gaa ggg tcc cag gca gtt ct	aac ttc tcc ggg acc aca ca	
**Hes7**	SYBR	gaa gct gga gaa agc gga ga	tcc ctc aag tag ccc acg g	
**Irx3**	SYBR	aag aga gca aac gcg agc tg	cct ctc ctg cga gct cct c	
**Isl1**	SYBR	tgc gga ctg tgc tca acg	cat acc agg tcc gca agg tg	
**MafB**	SYBR	cag ctc cgt gcc ttc ttc tc	tgg gtc ttc ggt tca gtc g	
**Myt1**	SYBR	atc gtg gag gtt cgc tct ga	ctg gga gcg tga atc ctc at	
**Nepn**	SYBR	gca ggt cct caa cct cag ga	gcc ttg aaa tct aag gcc gag	
**NeuroD1**	Taqman	cca gcc cac tac caa ttt gg	ggg ttc tgc tca ggc aag aa	tgc ctg cag ctc aac cct cgg
**Ngn3**	Taqman	gac gcc aaa ctt aca aag	gtc agt gcc cag atg t	cct gcg ctt cgc cca caa ct
**Pdx1**	Taqman	Applied Biosystems #Mm00435565_m1		
**Smarca4**	SYBR	atc aac ggg cct ttc ctc a	gcc cag ttt gac agt gtc ga	
**Spink3**	Taqman	Applied Biosystems #Mm00436765_m1		
**Tm4sf4**	Taqman	Applied Biosystems #Mm00523755_m1		
**Tmem27**	SYBR	cca tgt cct gct ttg caa ca	acc aca aac cag aac gac acc	
**Wnt4**	SYBR	aca ggg ctt cca gtg gtc ag	gct acg cca tag gcg atg tt	
